# Evaluation of the truebeam machine performance check (MPC): mechanical and collimation checks

**DOI:** 10.1002/acm2.12072

**Published:** 2017-04-17

**Authors:** Michael P Barnes, Peter B Greer

**Affiliations:** ^1^ Department of Radiation Oncology Calvary Mater Hospital Newcastle Waratah NSW Australia; ^2^ School of Medical Radiation Sciences University of Newcastle Newcastle NSW Australia; ^3^ School of Mathematical and Physical Sciences University of Newcastle Newcastle NSW Australia

**Keywords:** linac quality assurance, machine performance check (MPC)

## Abstract

Machine performance check (MPC) is an automated and integrated image‐based tool for verification of beam and geometric performance of the TrueBeam linac. The aims of the study were to evaluate the performance of the MPC geometric tests relevant to beam collimation (MLC and jaws) and mechanical systems (gantry and collimator). Evaluation was performed by comparing MPC to QA tests performed routinely in the department over a 4‐month period. The MPC MLC tests were compared to an in‐house analysis of the Picket Fence test. The jaw positions were compared against an in‐house EPID‐based method, against the traditional light field and graph paper technique and against the Daily QA3 device. The MPC collimator and gantry were compared against spirit level and the collimator further compared to Picket Fence analysis. In all cases, the results from the routine QA procedure were presented in a form directly comparable to MPC to allow a like‐to‐like comparison. The sensitivity of MPC was also tested by deliberately miscalibrating the appropriate linac parameter. The MPC MLC was found to agree with Picket Fence to within 0.3 mm and the MPC jaw check agreed with in‐house EPID measurements within 0.2 mm. All MPC parameters were found to be accurately sensitive to deliberately introduced calibration errors. For the tests evaluated, MPC appears to be suitable as a daily QA check device.

## 1 Introduction

Daily quality assurance (QA) testing of linear accelerators (linacs) is standard radiotherapy practice. In 2009, the AAPM Task Group 142^1^ report was published to supersede the AAPM Task Group 40 for recommendations on linac QA. The TG‐142 report stipulates a daily linac QA program including mechanical testing including the laser localization, distance indicator, and collimator size indicator. On a monthly basis among other things, TG‐142 recommends testing the gantry and collimator readouts, jaw positioning, and MLC leaf position accuracy. If these monthly linac parameters could be quickly and accurately tested on a daily basis, then the recommendations of TG‐142 would be exceeded.

With the TrueBeam 2.0 platform Varian (Varian Medical Systems, Palo Alto, CA, USA) has released the machine performance check (MPC) application. MPC is a fully integrated image‐based tool for assessing the performance of the TrueBeam critical functions. MPC tests are broken into two categories: The beam constancy checks and the geometric checks. It is the geometric tests relevant to collimation and linac mechanical systems which are the focus of this study.

At the time of writing, there was only a single paper in the literature pertaining to evaluation of MPC. Clivio et al., 2015^2^ published work whereby the results of MPC were compared against other more standard QA techniques. In Clivio's study, both MPC and the standard QA tests were run together on 10 consecutive days. From this dataset, the mean and standard deviation (SD) was calculated for both MPC and standard QA measurements and compared. The short duration of the study does not allow for any assessment of long term stability and there is no measure of MPC sensitivity, both of these shortcomings are acknowledged by the authors.

It is the aim of this study to compare the MPC mechanical geometric checks against standard QA tests to provide the reader with a sense of how the MPC checks might compare to their standard QA tests. The study was performed over a longer period (4 months) than the Clivio study and provides an assessment of the MPC stability and sensitivity to drift of the linac systems being tested. Sensitivity is further examined by the use of deliberate changes to the MLC centerline offset and gap, offset of the collimator calibration and offset and change in calibration span of the gantry calibration. The study also attempts to provide standard QA results in a form that is directly comparable to the equivalent MPC test.

## 2 Methods

### 2.A Materials

All measurements in this study were performed on a single Varian TrueBeam 2.0 STx linac fitted with an aS1200 EPID and 6 degree of freedom couch. The aS1200 EPID utilizes a 43 × 43 cm^2^ panel with backscatter absorber plate between the detection panel and positioning arm. The detector matrix is 1280 × 1280 with a smaller 1190 × 1190 pixel region employed for Dosimetry (Integrated) imaging mode providing a 0.23 mm resolution when EPID is at 150 cm source to detector distance (SDD) as is used for the MPC geometric tests.

#### 2.A.1 MPC geometric checks

The MPC geometric tests utilize a series of kV and 6 MV images of the IsoCal phantom situated in a specific bracket on the IGRT couch top to assess: treatment/radiation isocenter size, coincidence of MV and kV isocenters, accuracy of collimator and gantry angles, accuracy of jaw and MLC leaf positions, and accuracy of couch positioning including pitch and roll. All measurements are highly automated and the user is simply required to setup the IsoCal phantom and bracket onto the treatment couch at position H2 and to beam‐on. For the geometric tests, the system makes all required motions automatically and beams on when all is in position. Images are automatically analyzed at the TrueBeam console and results are presented with a pass/fail criteria applied. Functionality for presenting trends in results is also available in the package.

### 2.B Measurement methods

#### 2.B.1 Repeatability

Short‐term repeatability of the MPC geometric tests was evaluated by taking five successive measurements and calculating the SD.

#### 2.B.2 Jaw position evaluation

The MPC check of jaw positioning is performed using an 18 × 18 cm field. Jaw edges are detected on the EPID and the result is calculated as the distance between the measured jaw edge and the center of rotation of the MLC, which is determined from a series of collimator rotated MLC defined fields. As such, the measurement is not influenced by the absolute positioning of the EPID panel.

The department has maintained an in‐house electronic‐portal‐imaging‐device (EPID)‐based linac QA program for approximately 10 years^3^, ^4^, ^5^ including an in‐house EPID check of symmetric jaw positions. The in‐house EPID linac QA program utilizes the EPID in integrated mode with detector at 100 cm SDD. Images are analyzed using an in‐house developed MATLAB script (The Mathworks Inc., Natick, MA, USA). For jaw positioning QA, the beam central axis is determined from the average field center of two 10 × 10 cm fields at 180° opposed collimator angles. The resulting center pixel position is dependent only on the EPID panel positioning and the focal spot position of the beam. Because of the collimator rotation the effect of jaw positioning is removed. Using the measured center pixel as reference the position of the field edges are measured from a 20 × 20 cm jaw defined field and compared to expected. The shared methodology between MPC and the departmental in‐house EPID allows a direct comparison.

On a daily basis, the department relies on the Sun Nuclear Daily QA3 (Sun Nuclear Corporation, FL, USA) model 1093 running software version 2.4.1.2 X and Y size and shift parameters to check jaw positioning. The QA3 is a 2D array of ionization chambers and diodes. Following alignment to crosshairs or lasers, data are acquired from a single 20 × 20 cm^2^ field at 100 cm source to surface distance (SSD) and are compared to baseline. The X and Y size and shift parameters refer to the position and size of the radiation field relative to the laser/crosshair used to setup the device. The edge of the radiation field is detected using an array of diodes positioned across the beam penumbra. Analysis of the radiation field size and shift parameters in combination allows assessment of each individual jaw position and these were then compared against MPC.

The traditional method of measuring jaw positions relative to the crosshairs using the light field and graph paper was also performed for comparison to MPC. For jaw positioning, MPC uses a tolerance of ± 1 mm for the X jaws and ± 2 mm for the Y jaws. Having two different tolerances is likely due to mechanical reasons and the fact that the Y jaws are situated further from isocenter. The departmental monthly QA test uses a ± 1 mm tolerance and TG‐142 recommends monthly ± 2 mm for symmetric jaws, which is the tolerance used for the Daily QA3 check.

#### 2.B.3 MLC position evaluation

The MPC MLC test utilizes a static MLC “comb” pattern whereby alternating leaves are set at either 5 mm or 35 mm. The leaf positions are measured using EPID and the position of each leaf is determined relative to the collimator rotation axis determined from a series of collimator rotated MLC fields. MPC reports both the mean and maximum measured offset for each MLC bank. As such, the measurement is not influenced by the absolute positioning of the EPID panel.

The departmental routine QA of the positional accuracy of the MLC is based upon the Picket Fence method^6^ using the EPID and analyzed using an in‐house developed MATLAB script.^5^ Similar to the MPC MLC test, the departmental Picket Fence relates the position of the MLC leaves to the center of collimator rotation axis. Because of the similarity in methodology and detector a direct comparison can be made between MPC and the departmental Picket Fence test. From the Picket Fence image the width and position of each Picket are reported for each leaf pair. From these two parameters, the individual positions of the opposing MLC leaves can be determined and then using the respective Picket that corresponds most closely with each individual leaf in the MPC MLC comb pattern a comparison can be made between the two methods. Both MPC and the Picket Fence test utilize a ± 1 mm tolerance.

#### 2.B.4 Collimator

The MPC collimator rotation offset is derived from measurements of an MLC defined open field at five collimator angles. The reported result is determined as the maximum deviation of the nominal versus measured rotation angle observable through the edge of the MLC leaves. Tolerance is set at ± 0.5°. By utilizing the MLC leaves any skewing of the MLC will influence the result, but the absolute positioning reproducibility of the EPID panel will not influence the result.

The departmental Picket Fence analysis software includes a measure of the total skew in the Pickets caused by MLC bank skew, EPID panel skew and collimator rotation inaccuracy. The methodology is presented in detail in Rowshanfarzad et al., 2012.^5^ The method is based upon measurement of the angle of the Pickets compared to both the EPID image and also to the interleaf leakage apparent on the image between MLC leaves. Having these two different references allows the MLC skew to be isolated from the collimator rotation offset and EPID panel skew. For consistency of method, it is the total skew that is compared against MPC.

The traditional test used by the department to check collimator readouts is to use the gantry swing test. This test determines the collimator angle at which the cross wire projection on the floor is parallel to the axis of gantry rotation having verified at commissioning the cross wire parallelism/orthogonality with jaws and MLC. The other cardinal collimator angles are checked with gantry rotated to 90° or 270° and using a digital spirit level which is rated to ± 0.01° accuracy (Digi‐Pas 2‐Axis Precision Digital level, DWL3000XY, DIGIPAS, LLC, USA) against the collimator faceplate (again parallelism of the collimator faceplate to the crosshair is verified at commissioning). Tolerance is ± 0.5 degrees. MPC reports the greatest deviation over the five collimator angle measurements so for comparison in this study the mechanical QA method cardinal angle with the greatest deviation from nominal was compared against MPC.

#### 2.B.5 Gantry

MPC reports two results for the gantry. Firstly, the MPC gantry absolute measure reports on the coincidence of the couch vertical axis with the central beam axis at gantry readout of zero degrees. This is done using two successive MV images of the IsoCal phantom at different couch vertical heights. The relative positioning of the phantom in the two images is used to calculate the angle between beam axis and couch vertical axis. As the EPID is not moved between the two images then its absolute position does not influence the result. The measurement is performed for both the lateral and longitudinal planes with the worse‐case value reported. The lateral measurement would be influenced by the verticality of couch travel as well as the accuracy of the gantry readout. The longitudinal measurement would be influenced by the verticality of couch travel as well as gantry sag. The MPC gantry absolute measure provides a measure of the accuracy of the gantry position at G0 and hence the accuracy of the absolute calibration of the gantry readout encoder.

Secondly, the MPC gantry relative check utilizes MV images of the IsoCal phantom at eight gantry angles (45° separated) and reports the maximum offset between the angle determined by the MV imaging system and the nominal gantry angle.^7^ This measure will be influenced by linearity and span of the gantry primary encoder and by any changes in beam central axis with gantry rotation. The EPID panel absolute positioning will not influence the measurement.

The traditional method the department uses to test gantry angle readouts is to use a calibrated spirit level on the collimator faceplate at the cardinal gantry angles. Tolerance is ± 0.5°. For comparison to MPC gantry absolute, the difference from nominal gantry angle at G0 was compared, while for the gantry relative comparison, the maximum variation in measured angle from the expected 90° for two adjacent cardinal angles is compared.

#### 2.B.6 Sensitivity of MPC to linac miscalibration

The MPC gantry, collimator, MLC, and jaw positioning checks were each tested for sensitivity to miscalibration of the relevant linac parameter. Firstly, the collimator was deliberately miscalibrated using the digital spirit level. Successive offsets in the collimator rotation calibration were induced based upon the spirit level to the order of ± 0.5°. MPC was run premiscalibration, after each miscalibration and finally at the end when the calibration was returned to optimal. The measured changes in MPC were compared to the expected from the spirit level.

The sensitivity of the MPC gantry tests was examined by inducing miscalibration to the gantry angle in two ways. Firstly, a systematic offset of 0.5° was introduced into the calibration alternately in both directions and secondly the span of the calibration was miscalibrated by 2° (0.55%) alternately smaller and larger. MPC was performed before and after each miscalibration and measured changes in MPC gantry absolute and gantry relative were compared to the expected from the spirit level.

The sensitivity of the MPC MLC checks was tested by inducing changes to the MLC calibration. To do this, the MLC centerline offset was changed from 0 mm to + 1 mm. MPC was performed pre and post change and the change in MPC mean and max offset was recorded for both MLC banks. The expected effect of changing the centerline offset would be to shift the position of both MLC banks by the set amount in the same direction. After resetting the centerline offset back to 0 mm, the MLC centerline gap was changed from 0 mm to + 1 mm and again the measured change in MPC MLC mean and maximum offset was recorded. By changing the centerline gap by + 1 mm, the effect would be to move the MLC banks away from each other by half the set amount each.

The sensitivity of the MPC jaw positioning was investigated using deliberate miscalibration of jaw positions based upon the traditional method using the light field and graph paper. The jaws were systematically adjusted by ± 2 mm for the Y jaws and ± 1 mm for the X jaws and recalibrated thus. The measured change in MPC and in‐house EPID QA were recorded.

## 3 Results

### 3.A Repeatability

**Figure 1:**
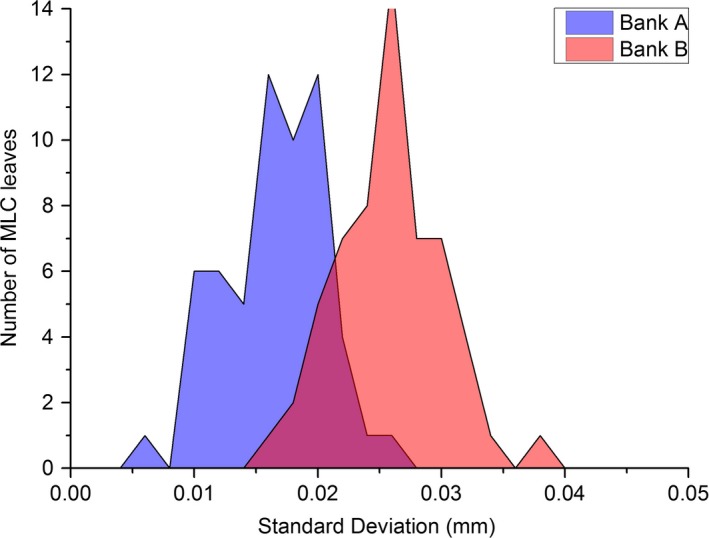
MPC MLC repeatability histogram for both MLC Banks A and B.

The results of Table 1 show how repeatable each of the MPC geometric tests were across five successive measurements. The MLC results of Table 1 are for the maximum and mean offsets across each MLC bank. MPC also reports results for each MLC leaf individually and the spread in repeatability across the leaves for each bank is presented in histogram form in Fig. 1.

**Table 1:** Short‐term repeatability of the MPC geometric tests based upon five successive measurements

Test	Standard deviation
Collimator	0.01°
Gantry	
Absolute	0.01°
Relative	0.00°
Jaw	
X1	0.02 mm
X2	0.01 mm
Y1	0.08 mm
Y2	0.04 mm
MLC	
Max offset Bank A	0.02 mm
Max offset Bank B	0.02 mm
Mean offset Bank A	0.02 mm
Mean offset Bank B	0.02 mm

The repeatability results of Table 1 show that for all MPC tests, the methods are repeatable to within 0.1 mm or 0.01° for all parameters at 1 SD. The results of Fig. 1 show that the repeatability of the individual MLC leaf positions is within 0.04 mm at 1 SD and that bank A is systematically more repeatable than bank B by approximately 0.01 mm.

### 3.B MLC positioning

**Figure 2:**
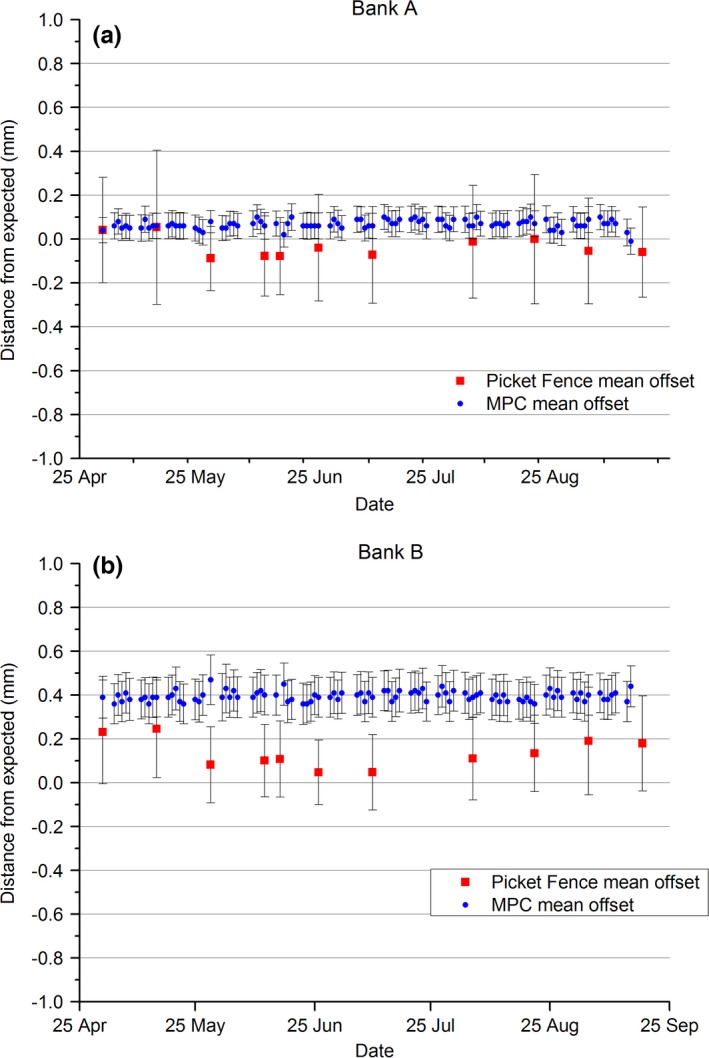
MPC MLC and in‐house MLC Picket Fence test results mean difference from expected across the whole MLC bank. Error bars are for 1 SD across the bank. (a) Bank A, (b) Bank B.

The results of Fig. 2 demonstrate the agreement between the MPC MLC mean offset and the equivalent mean offset calculated using the in‐house Picket Fence method. The error bars for each method are calculated based upon the spread of results across all the leaves measured in the bank. Figure 2(a) shows that the MPC MLC mean offset results were always within 1 SD of the Picket Fence mean results and that both methods indicated that within uncertainty on average the MLC Bank A was ideally calibrated. Figure 2(b) shows that for MLC Bank B, the MPC mean offset was not within 1 SD of the Picket Fence results. The Picket Fence results suggest that on average the MLC Bank B deviated from optimal by 0.13 mm, while MPC suggests deviation of 0.4 mm.

**Figure 3:**
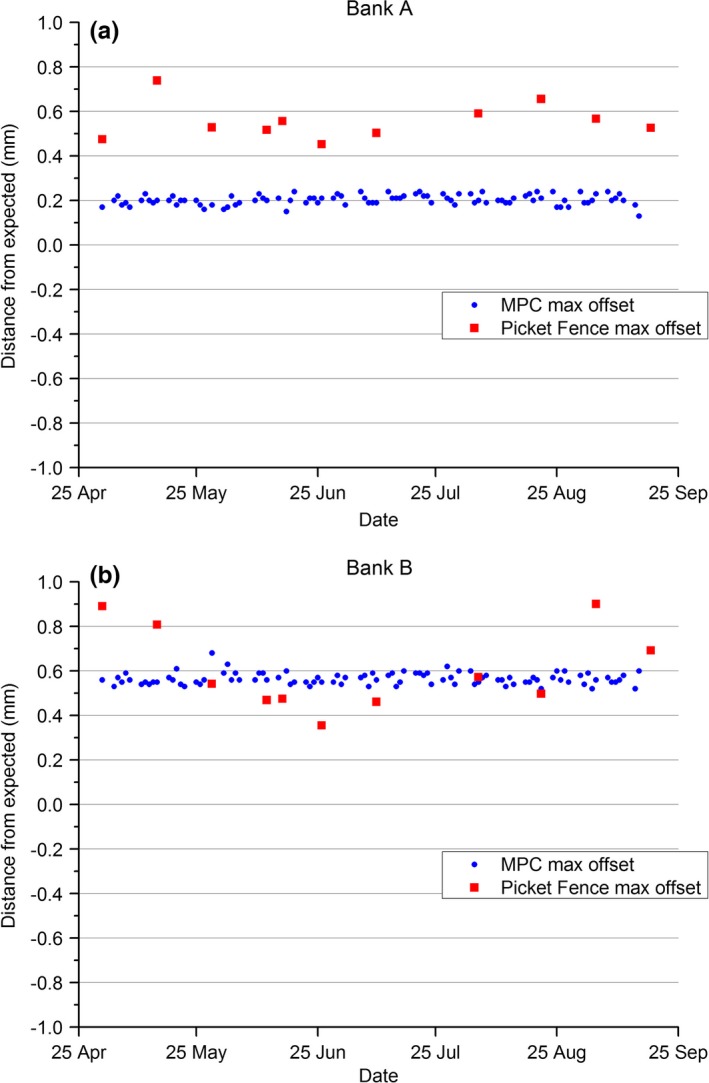
MPC MLC and in‐house MLC Picket Fence test results maximum difference from expected across the whole MLC bank. (a) Bank A, (b) Bank B.

The results of Fig. 3 show how the MPC MLC max offset value corresponds to the equivalent Picket Fence value. Figure 3(a) indicates that the MPC MLC max offset value is stable for MLC Bank A at 0.2 mm, while the Picket Fence result has both greater variation and a systematically higher result at approximately 0.55 mm. For MLC Bank B, Fig. 3(b) shows the greater variation of the Picket Fence result, but both Picket Fence and MPC average approximately 0.6 mm.

#### 3.B.1 MPC MLC sensitivity to miscalibration

Table 2 shows that for a nominal shift of + 1 mm in the MLC centerline offset, the measured change in MPC mean offset was within 0.05 mm of expected for both banks and that this was within 1 SD across the leaves within the bank. For a change in MLC centerline gap of + 1 mm, Table 2 shows that the MPC mean offset values recorded were accurate to within 0.07 mm.

**Table 2:** MPC MLC sensitivity to both an offset in the MLC centerline offset of + 1 mm and to a change in the MLC centerline gap of + 1 mm. Results presented as the difference from expected in the MPC mean offset. Standard deviation relates to the results across the individual leaves within the bank

Change in calibration	Bank A	Bank B
	Mean ± 1 SD	Mean ± 1 SD
+1 mm offset	0.02 ± 0.09	−0.05 ± 0.09
+1 mm gap	0.01 ± 0.09	−0.07 ± 0.09

### 3.C Jaw positioning

**Figure 4:**
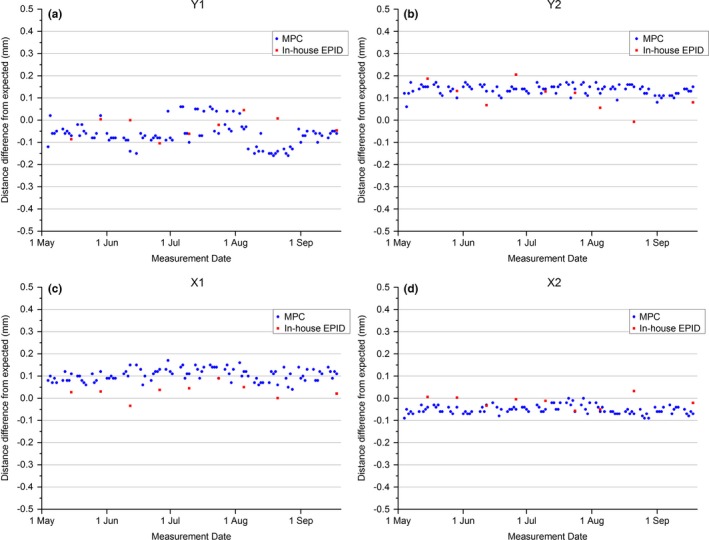
MPC and in‐house EPID measured jaw position distance from collimator rotation axis difference from expected. (a) Y1, (b) Y2, (c) X1, and (d) X2.

**Figure 5:**
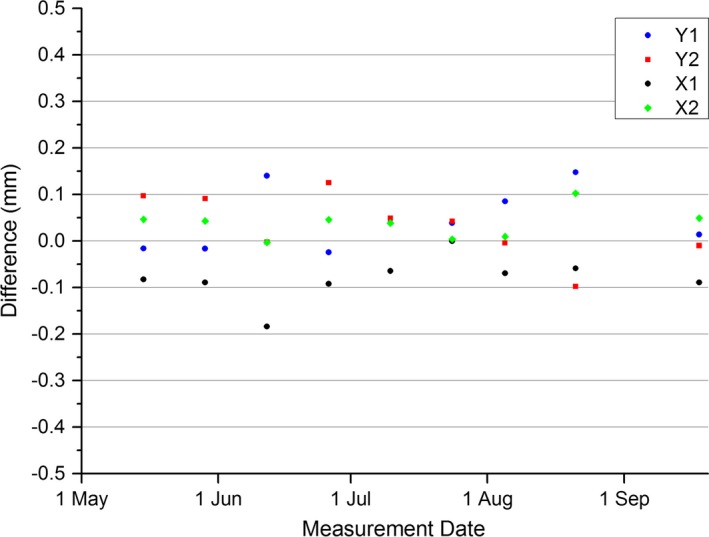
MPC and in‐house EPID measured jaw position agreement (in‐house EPID QA–MPC).

Figure 4 shows the measured MPC jaw positions against the jaw positions measured with the in‐house EPID method. The results show that the MPC measurement is within ± 0.2 mm of optimal for all measurements. As there is no obvious trend in the data for either method with any of the jaws then a mean and SD is meaningful. These data are presented in Table 3. The measured difference between the in‐house EPID method and the MPC measurement on the same day is presented in Fig. 5. Figure 5 shows that for all measurements, the in‐house EPID method agreed with MPC to within 0.2 mm. Analysis using the t‐test shows that MPC is in statistical agreement with the in‐house EPID method for the Y jaws, but not for the X jaws (Y1: *P* = 0.08, Y2: *P* = 0.23, X1: *P* << 0.01, X2: *P* < 0.01).

**Table 3:** Mean jaw position difference from expected (mm) for MPC (n = 95), QA3 (n = 91), and in‐house EPID measurements (n = 9). (Mean ± 1 SD)

Jaw	MPC	QA3	In‐house EPID
X1	0.11 ± 0.03	−0.19 ± 0.47	0.03 ± 0.03
X2	−0.05 ± 0.02	−0.18 ± 0.48	−0.01 ± 0.03
Y1	−0.06 ± 0.06	−0.54 ± 0.32	−0.03 ± 0.05
Y2	0.14 ± 0.02	0.12 ± 0.37	0.11 ± 0.07

**Figure 6:**
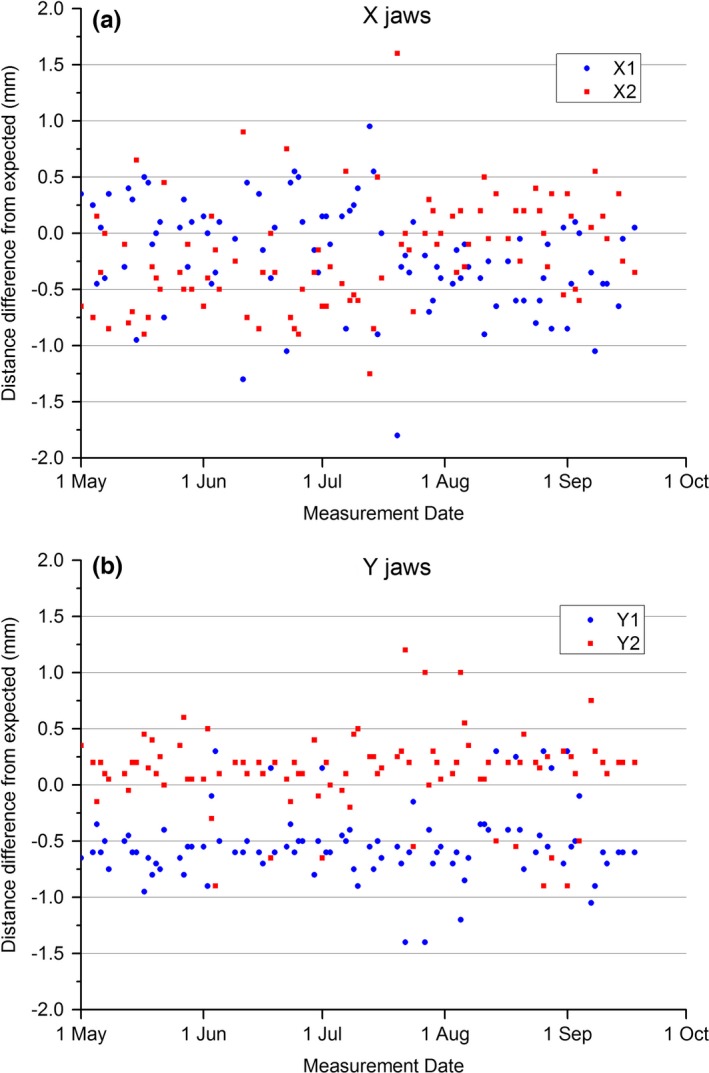
QA3 measured jaw positions. (a) X jaws and (b) Y jaws.

Figure 6 shows the QA3 measured jaw positions derived from the field shift and field size parameters over the same time period as the MPC measurements presented in Fig. 5. Comparison between the two Figures indicates greater variability in the QA3 measurements compared to MPC and also a larger variation from the nominal value. As there is no obvious trend in the data, the mean and SD data for the QA3 were included in Table 3.

The results of Table 3 show that the MPC mean value was within 1 SD of the QA3 mean for all jaws except the Y1 jaw which was within 2 SD. The QA3 had much greater SD then either MPC or the in‐house EPID indicating greater variability. The t‐test shows statistical agreement between MPC and QA3 for the Y2 jaw only (Y1: *P* << 0.01, Y2: *P* = 0.59, X1: *P* << 0.01, X2: *P* < 0.01).

Over the period of the study, the jaw positions were also tested using the traditional method using the light field and graph paper placed at isocenter. The method allows only 1 mm measurement resolution and this was insufficient to make any meaningful comparison with MPC.

#### 3.C.1 MPC jaw position sensitivity to miscalibration

The results of Table 4 show that both the MPC and in‐house EPID measured changes in jaw positioning are not always as expected. Differences from expected up to 0.81 mm were recorded for MPC and up to 0.68 mm were recorded for the In‐house EPID. However, agreement between MPC and in‐house EPID was always within 0.13 mm.

**Table 4:** MPC Jaw positioning sensitivity to 2 mm changes in Y jaw calibration and 1 mm change to X jaw calibration

Calibration change	Difference from expected (mm)		Difference (mm)
	MPC	In‐house EPID	In‐house EPID − MPC
Y1			
+2 mm	−0.08	−0.05	0.03
−2 mm	0.81	0.68	−0.13
Back to 0 mm	0.06	0.13	0.07
Y2			
+2 mm	−0.58	−0.54	0.04
−2 mm	−0.09	0.02	0.11
Back to 0 mm	−0.24	−0.14	0.10
X1			
+1 mm	−0.10	−0.16	−0.06
−1 mm	−0.04	−0.02	0.02
Back to 0 mm	−0.16	−0.13	0.03
X2			
+1 mm	−0.14	−0.16	−0.02
−1 mm	−0.28	−0.02	0.11
Back to 0 mm	−0.19	−0.13	0.02

### 3.D Collimator

**Figure 7:**
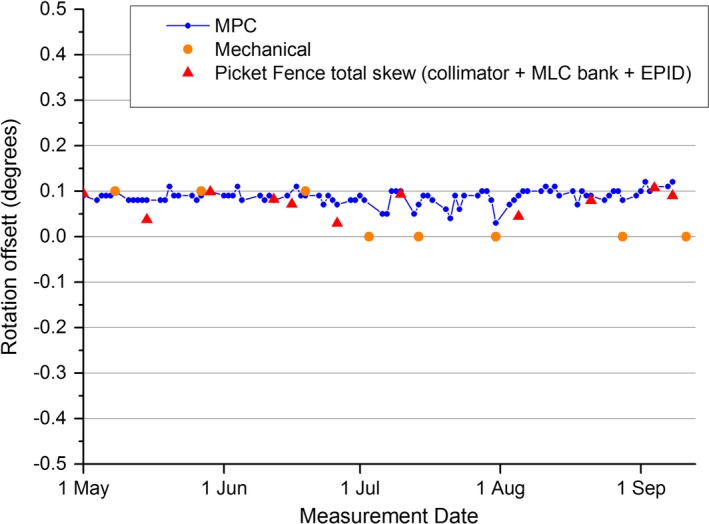
MPC measured collimator rotation offset plotted over a 4‐month measurement period alongside the measured Picket Fence total skew which includes collimator rotation offset, MLC bank skew, and EPID panel skew. Also included are standard mechanical QA results for collimator readout accuracy at collimator = 0.

**Figure 8:**
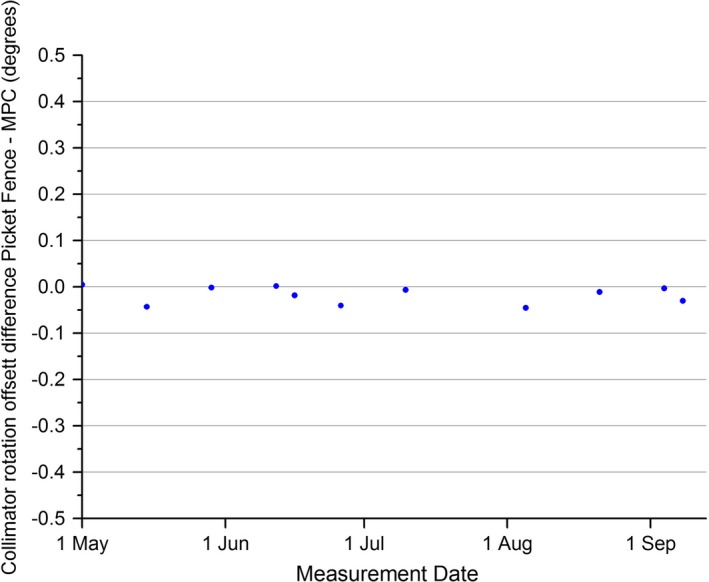
Measured difference between MPC collimator rotation offset and the Picket Fence total skew.

**Figure 9:**
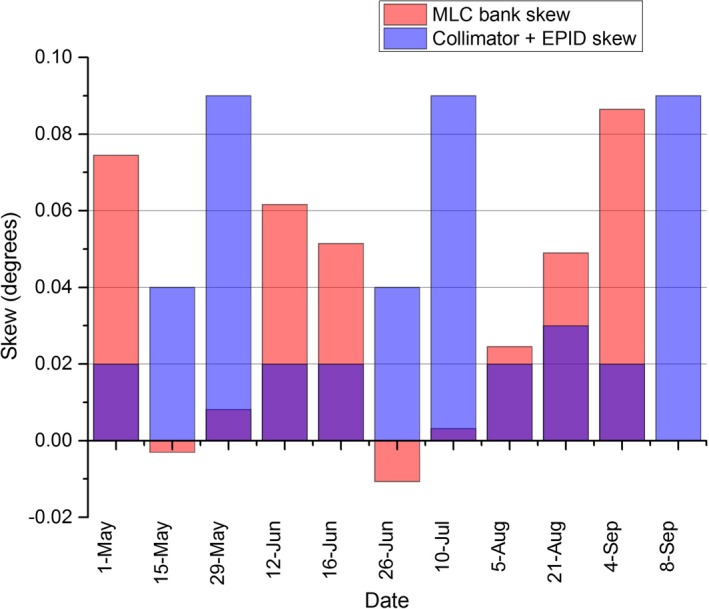
The Picket Fence total skew results of Fig. 7 broken into the contributing components of MLC bank skew and collimator rotation + EPID skew.

The results of Fig. 7 show the MPC collimator rotation offset and the Picket Fence total skew parameters were always within 0.12° of nominal. The mechanical QA method is limited by the collimator readout being limited to tenth of a degree resolution so over the period, the measured value was always 0.0 or 0.1 degrees difference from nominal. As there is no apparent trend in the MPC or Picket Fence data then calculating a mean and SD is meaningful. The MPC mean was calculated over the period to be 0.087° ± 0.016 (1 SD), while the Picket Fence mean was calculated to be 0.075° ± 0.026 (1 SD) meaning that the MPC mean was within 1 SD of the Picket Fence mean. The t‐test shows statistical agreement between the two methods (*P* = 0.16). The measured differences between the two methods are plotted in Fig. 8 with the greatest difference at 0.0455°. The mechanical QA mean was measured to be 0.038 ± 0.052 (1 SD). The MPC and Picket Fence results of Fig. 7 include the influence of MLC bank skew. As MPC reports the result as collimator rotation offset it might be more meaningful to separate out the MLC bank skew. This is done within the Picket Fence method and the contributions from individual components are presented in Fig. 9. Figure 9 demonstrates that the MLC bank skew contributes significantly to the measurement.

#### 3.D.1 MPC collimator sensitivity to miscalibration

The results of Table 5 show that for an offset in the collimator rotation calibration of the order of magnitude comparable to the MPC tolerance (± 0.5°), the measured change in MPC agrees with the measured change on the spirit level to within 0.07°.

**Table 5:** Measured changes in collimator rotation angles with 0.5° miscalibrations (degrees)

Collimator calibration	Spirit level	MPC	Difference (spirit level − MPC)
−0.5	−0.53	−0.60	0.07
+0.5	0.47	0.54	−0.07
0 (final)	0.03	−0.02	0.05

### 3.E Gantry

**Figure 10:**
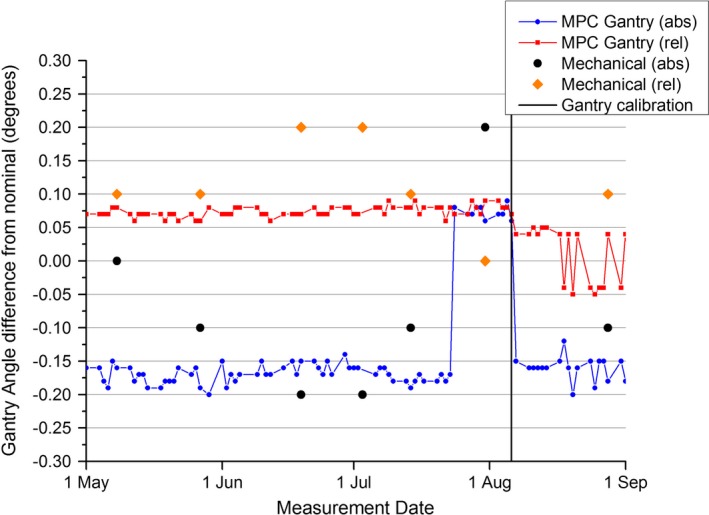
Measured gantry difference from nominal for MPC absolute, MPC relative, mechanical absolute, and mechanical relative over the 4‐month measurement period. Also, indicated is the gantry angle readout calibration performed on August 6.

The results of Fig. 10 show that from the beginning of May until the July 23, both the MPC relative and absolute measurements were relatively stable. In this period, MPC relative gantry measured a mean of 0.07 ± 0.07° (1 SD) and the MPC gantry absolute measured a mean of −0.17 ± 0.07° (1 SD). At July 23, there was an overnight jump in the gantry absolute results, which was thereafter stable with a mean of 0.07 ± 0.01 degrees (1 SD). This is a statistically significant change (t‐test: *P* << 0.01).

During the periods before and after the jump observed on the July 23 in the gantry absolute data, there was no statistical difference in the gantry relative measurement according to the t‐test (*P* = 0.06). In this period, the gantry relative had a mean of 0.08 ± 0.01° (1 SD). At August 6, the gantry readouts were recalibrated at which point the gantry absolute results returned to a mean of −0.16 ± 0.02° (1 SD), which is statistically equivalent to before the jump on the July 23 (t‐test: *P* = 0.12). In the same period after the gantry readout calibration, the gantry relative measurement changed to a mean of 0.01 ± 0.04° (1 SD) and was no longer in statistical agreement to before the recalibration (t‐test: *P* << 0.01). In this period, the results are seen to oscillate about zero, which is a known behavior (Varian MPC user guide p39^7^).

The relatively coarse resolution of 0.1° for the mechanical QA gantry measurements makes meaningful comparison with MPC difficult. However, Fig. 10 does appear to show mechanical results tracking the MPC results in both relative and absolute cases. The mechanical absolute measurement agrees with MPC gantry absolute within measurement resolution before the jump on July 23 for four of the five measurement points. After July 23, when the MPC gantry absolute measurements jumps the mechanical measurement also jumps to the order of 3.5 times the measurement resolution and then returns back after the gantry recalibration on August 6. The mechanical gantry relative measurement agreed with MPC gantry relative to within twice measurement resolution over the entire measurement period.

#### 3.E.1 MPC gantry sensitivity to miscalibration

Table 6 shows that when the gantry angle is miscalibrated by an offset up to 0.5°, the MPC gantry absolute measure is in agreement with the digital spirit level to within 0.05° and that the MPC gantry relative measure is insensitive to such a miscalibration. Table 7 shows that for a 2° miscalibration in the gantry calibration span, there is insignificant change in the gantry absolute measure. However, the gantry relative measure changed from initial by 0.79° and 0.88°, respectively.

**Table 6:** MPC gantry tests sensitivity to 0.5° offset miscalibrations (degrees)

Collimator calibration	Spirit level	MPC gantry absolute	MPC gantry relative	Difference (spirit level – MPC absolute)
0 (initial)	−0.11	−0.11	0.05	0.00
−0.5	−0.63	−0.64	0.05	0.01
+0.5	0.34	0.29	0.05	0.05
0 (final)	0.02	−0.01	0.05	0.03

**Table 7:** MPC gantry tests sensitivity to 2° span miscalibrations (degrees)

Gantry calibration span (degrees)	MPC gantry absolute	MPC gantry relative
360 (nominal)	−0.02	0.05
358 (shorter)	−0.13	0.84
362 (larger)	−0.10	0.93
360 (nominal)	−0.14	−0.05

## 4 Discussion

### 4.A Repeatability

The repeatability results of Table 1 are well inside the tolerances for all tests indicating that the tolerances are meaningful in that recorded fails are distinguishable from day to day variation. Figure 1 indicates that the positioning of MLC leaves in Bank A are in general more repeatable than those in Bank B. However, the difference in the means of the two banks of 0.01 mm is insignificant.

### 4.B MLC Positioning

The measurement resolution of the EPID at 150 cm SDD of 0.23 mm is well within the tolerances for the MLC and jaw positioning of 1 mm. As such, the EPID resolution is considered sufficient for both the MLC and jaw positioning tests.

Explaining the measured differences between MPC and the in‐house Picket Fence analysis for MLC position testing requires an understanding of where the two methods differ. Both methods have commonality of detector (EPID) and of spatial referencing to the center of collimator rotation, and hence no dependence on detector positioning. The methods differ in that the collimator rotation axis for MPC is defined in each measurement session, whereas the Picket Fence references to an annual measurement. While the closest Picket within the Picket Fence image was chosen for comparison to MPC, the positions were not exactly the same. For MPC, the leaf positions are 5 mm and 35 mm from central axis and these were compared to the Picket Fence at leaf positions 0 mm and 40 mm, respectively from central axis. Also, to determine the individual leaf positions from the Picket Fence image, the peak position parameter was used along with the measured gap width to then determine the position of the individual MLC leaves. Because the Pickets are created by 1 mm overlaps in the MLC defined subfields, this means that the Pickets are made up of overlapping MLC penumbra. This means that when the overlap is increased, the peak height increases and vice versa. This makes it difficult to measure the gap width directly and hence the reported gap width parameter is determined by modeling the measured change in full‐width‐half‐maximum against deliberate known changes to the gap width. This methodology is overly complicated compared to MPC where the leaf position is determined as the 50% intensity point on a single MLC penumbra. Inaccuracies in the Picket Fence gap width modeling are expected to be the dominant source of variation between the measured Picket Fence and MPC MLC positions. When considering the differences between the MPC and Picket Fence methods all favor increased accuracy with MPC and this is potentially borne out in the reduced measurement to measurement variation in the MPC results and the smaller deviation in results across the leaf banks indicated by the smaller error bars in Fig. 2 compared to Picket Fence.

The results of Table 2 indicate accurate sensitivity of MPC MLC mean offset to changes in MLC position. For both the gap and offset calibration changes, MPC reported the result within 0.1 mm of expected. Such a deviation is clinically insignificant.

The MPC MLC results suggest that MPC is suitably accurate and sensitive as a daily QA of the MLC position. Considering that TG‐142 recommends a qualitative Picket Fence style test on a weekly basis then MPC would appear to exceed this requirement.

### 4.C Jaw positioning

The results in Table 3 indicate greater variability in QA3 measured jaw positions than either MPC or in‐house EPID and also a larger systematic shift in the result. The systematic shift in the result associated with QA3 could be explained by the fact that the QA3 setup is to the crosshairs, while the MPC and in‐house EPID methods reference to the center of collimator rotation and hence are independent of EPID positioning. Any offset in the light source or crosshair or user misalignment would manifest as a systematic shift and could partially explain the QA3 Y jaw results of Table 3 as the mean value is negative for Y1 and positive for Y2 indicating a systematic shift in the same direction for both jaws. Another possible contribution for such a shift would be a shift in the beam focal spot position and this is supported by the negative mean values for Y1 and positive mean values for Y2 in the MPC and in‐house EPID results. The negative mean values for both the X1 and X2 QA3 results in Table 3 indicate a field magnification discrepancy. This could be explained by a systematic error in setting the SSD to the QA3. No such magnification issue is supported by the MPC or in‐house EPID results.

In comparing the QA3 to the MPC, it should be noted that the QA3 recommended tolerance on the parameters used to calculate the jaw positions is ± 2 mm, which is in alignment with the AAPM TG‐142^1^ recommendations, whereas the MPC tolerance is 0.5 mm. Over the measurement period of this study, no method indicated a fail in jaw position including the graph paper method.

The differences in both MPC and in‐house EPID jaw position to the expected (up to 0.81 mm) when a miscalibration of the jaw up to 2 mm is introduced reflects more on the inaccuracy of using the light field to calibrate and test the jaws rather than inaccuracy in the EPID‐based measurement methods. Calibrating the jaws with the light field and graph paper first requires that the light source and crosshairs are centered on collimator rotation. This is the first source of uncertainty, which is complicated on the TrueBeam linac because there are two light sources which the linac utilizes interchangeably and translate into position on the same assembly as the monitor chamber. The mirror is located on the carousel, which also has a translation axis of motion. Larger uncertainty in the light source jaw calibration procedure is the graph paper resolution of 1 mm and the subjectivity in identifying the 50% point on the light field penumbra.

The agreement to within 0.13 mm between MPC jaw positioning and in‐house EPID for a nominal change in jaw position of 2 mm indicates agreement between the two methods. This is expected as the two methods have a common methodology in that they both reference to a center of collimator rotation axes and then detect jaw positions on a wider field using the 50% point on the penumbra as measured on EPID. The small differences that are observed could be due to jaw positioning repeatability or the fact that the in‐house EPID uses a 20 × 20 cm field size, while MPC uses an 18 × 18 cm field size so that if there is a nonlinearity in the jaw calibration, these two different measurement points may give a different result. To put the results into context, the maximum difference between MPC and in‐house EPID of 0.13 mm is clinically insignificant.

### 4.D Collimator

The statistical agreement between the MPC and Picket Fence collimator rotation methods suggest accuracy of MPC. The 0.1° limit of resolution of the mechanical method renders this method a little meaningless in comparison to MPC. The maximum difference recorded between MPC and Picket Fence methods was 0.046°, which demonstrates that over the measurement period, there were no clinically significant variations between MPC and Picket Fence. The measured accuracy MPC to a ± 0.5° offset in collimator rotation demonstrates that significant error would be detected. As such, MPC is judged to provide a suitable routine test of collimator rotation accuracy.

The nature of the MPC collimator rotation test, whereby the MLC banks are used as the reference for collimator rotation adds an extra variable to the measurement in the form of MLC bank skew. Figure 9 suggests that the MLC bank skew can contribute significantly to the overall measurement. Although this contribution is clinically negligible, it could be isolated and its value reported using the method used in the Picket Fence analysis and hence an extra linac parameter would be tested thus improving the MPC test suite.

### 4.E Gantry

The results of Fig. 10 show that the MPC gantry absolute measure agrees well with spirit level measurements of gantry angle at G0. Figure 10 and Table 6 suggest that the MPC gantry absolute result rather than the gantry relative result is sensitive to an offset miscalibration of the gantry angle encoder. On the July 23, there was an overnight jump in the MPC gantry absolute readout by 0.24°. Upon investigation, it was found that the day before the jump service engineers were investigating an SF_6_ gas leak on the linac in the vicinity of the primary gantry encoder. It is surmised that the encoder must have been accidentally bumped or some other way inadvertently adjusted during the service. The MPC gantry relative measure was relatively insensitive to this change.

On the TrueBeam system, the gantry encoder is calibrated using two measurements at G180 degrees (gantry head down) with the gantry approaching from opposite sides. The first miscalibration performed in this experiment was a systematic offset and the span of the encoder was not altered. This may explain why the gantry absolute measure was sensitive to the miscalibration while the gantry relative was not. The gantry absolute measure compares the angle of the beam axis at G0 to the axis of couch vertical motion. If the gantry encoder is miscalibrated, then the beam axis at G0 will not be vertical and hence the system will be sensitive to the miscalibration. However, if there is a problem with the encoder span or linearity then the single point nature of the test may render it insensitive to this type of problem. The gantry relative measure on the other hand compares the MV images of the Isocal phantom at eight different gantry angles. As the phantom has a known geometry then relative angles can be calculated from the images with the greatest measured deviation from expected reported. This test should be sensitive to changes in gantry calibration span or encoder nonlinearity. This is born out with the span miscalibration experiment where the gantry absolute measure was found to be insensitive and the gantry relative sensitive to a 2° change in span. Considering that the gantry calibration procedure is performed with calibration points at G180 from both directions then the G0 point becomes the midpoint between these calibration points and hence when the calibration is offset equally in both directions for span miscalibration then it could be expected that the midpoint would not be affected and gantry absolute is insensitive. The effect on gantry relative was significant. The 2° miscalibration resulted in a change in gantry relative that was over twice the magnitude of the allowed threshold.

The MPC gantry absolute test will be influenced by changes in the gantry encoder calibration, the couch vertical travel axis, and changes in gantry sag. If the results were presented for both lateral and longitudinal directions then the lateral measurement would be a more pure measure of gantry calibration, while the longitudinal measure would provide a measure of gantry sag. The accuracy of couch vertical travel would influence both measurement for both directions and hence the MPC gantry absolute measure should be considered only as an indicator for further investigation.

The combination of gantry absolute and gantry relative tests together in MPC provide robust testing the gantry accuracy of the gantry positioning system. The gantry absolute test provides a check of any offset in the calibration while the gantry relative check tests the encoder span and linearity. Together these two should ensure accurate gantry angles over the full allowed range.

While the testing performed in this study suggests that MPC could make a valuable addition to a department's linac QA program, it must be cautioned that MPC should be treated like any QA system in that it should be thoroughly commissioned to the extent that the department is satisfied as to its utility. Like any clinical QA device, an ongoing QA program should be put in place to ensure ongoing accuracy. Running MPC in parallel with existing daily QA methods in the department as well as sensitivity measurements to commonly expected faults should be included in commissioning of MPC. Ongoing QA should include benchmarking against monthly and annual QA tests as well as routine QA of the EPID and OBI systems, which should include regular updates to the EPID dark field and pixel correction map and regular performance of IsoCal verification to ensure MPC accuracy.

As potential further work to this study, the next step for evaluation of MPC could be a multicenter study to evaluate the variation in MPC performance across multiple linacs. As part of such a study, the response of each MPC test could properly be evaluated. To do this, the sensitivity experiments performed in this study could be repeated on multiple linacs and over a range of miscalibrations to determine the linearity or otherwise of the MPC response. For example, the MLC centerline offset miscalibration experiment could be performed with values of −2, −1.5, −1.0, −0.5, 0.5, 1.0, 1.5, and 2.0 mm and the response of the MPC MLC test evaluated. If the department's standard MLC positional test was performed in parallel to MPC, then the accuracy of the MLC centerline offset could also be evaluated.

## 5 Conclusion

The MPC MLC mean and maximum offset, jaw positioning, collimator rotation, and gantry absolute and relative parameters have been evaluated for short‐term repeatability, sensitivity to induced changes, and compared against the methods currently in use in the department's linac QA program over a period of 4 months. The MPC MLC tests have all been shown to be accurate and sensitive within clinical significance and to exceed the recommendations of TG‐142 for daily linac QA. The results indicate that the MPC tests covered in this study could be used for linac daily QA as long as the system is properly commissioned, its limitations understood and the individual department satisfied as to its utility. A robust ongoing QA program for MPC is also required.

## Conflict of Interest

The authors declare no conflict of interest.
